# Strategic foresight and AI readiness: mediating roles of digital transformation capability and students’ perceptions of institutional agility

**DOI:** 10.3389/frai.2026.1928999

**Published:** 2026-09-17

**Authors:** Bright Asare, Sadri Alija, Yarhands Dissou Arthur, Francis Ohene Boateng

**Affiliations:** 1 Akenten Appiah-Menka University of Skills Training and Entrepreneurial Development, Kumasi, Ghana; 2 South East European University, Tetovo, North Macedonia

**Keywords:** AI readiness, digital transformation capability, institutional agility, mediating role, strategic foresight

## Abstract

**Purpose:**

The study aimed to investigate the mediating roles of digital transformation capability and students’ perceptions of institutional agility on the relationship between strategic foresight and AI readiness in higher education.

**Methodology/design/approach:**

The study adopted a cross-sectional SEM analysis research design. The study targeted university students with a sample size of 311 students. The target students were selected using a convenience sampling technique. The data collection for the study was conducted using an online questionnaire designed based on the study constructs. The data collated were then analyzed using structural equation modelling (SEM) run in Amos (ver. 23) to answer the hypothesized paths.

**Findings:**

The study results revealed that strategic foresight positively predicted AI readiness. Moreover, the study found that strategic foresight positively predicted digital transformation capability. In addition, the study found that digital transformation capability positively predicted AI readiness. Furthermore, the study found that digital transformation capability partially mediates the relationship between strategic foresight and AI readiness. Finally, the study found that students’ perceptions of institutional agility partially mediate the relationship between strategic foresight and AI readiness.

**Novelty/originality:**

Despite the increasing number of studies on artificial intelligence, very limited concentration has been paid to how strategic foresight influences AI readiness in higher education. This study did not just establish the effect of SFO on AI_R but also identified some intervening variables in this relationship, such as DTC and SPIAG.

## Introduction

One of the most disruptive technical forces changing higher education systems globally is artificial intelligence (AI). To improve teaching quality, research productivity, and administrative efficiency, universities are increasingly implementing AI-enabled technologies, such as learning analytics, intelligent tutoring systems, automated assessment tools, and generative AI applications (Bacalso et al., 2026). Higher education institutions (HEIs) are moving more quickly from traditional knowledge transmission models to data-driven, adaptive, and intelligent learning ecosystems as a result of this advancement.

The successful adoption and application of artificial intelligence (AI) in higher education is still quite uneven between institutions, despite the quick advancement of technology. While some universities show early AI integration and advanced digital maturity, many others still struggle with organizational and structural issues like inadequate digital infrastructure, a lack of preparedness for human capital, disjointed governance systems, and a lack of alignment between technological innovation and strategic planning. These differences imply that adopting AI is a complicated organizational change that calls for long-term institutional skill development and strategic alignment, rather than being solely a technology process (Abid, 2026).

AI readiness should be viewed as an organizational learning and capability-building process rather than a straightforward technology acquisition issue, according to recent research, where successful implementation outcomes are largely dependent on leadership, governance, and human-AI interaction (Dutta and Poyil, 2025). As a result, infrastructure, decision-making processes, and adaptable organizational capabilities that support long-term digital transformation must all be in line for institutions to be ready for AI.

First- and second-year students may not be directly involved in institutional decision-making; however, they are important stakeholders who experience and interact with university systems, policies, and practices on a regular basis. Through their engagement with academic programs, administrative procedures, digital learning platforms, student support services, and other institutional facilities, students develop an understanding of how institutional strategies and processes are implemented in practice. Therefore, the constructs examined in this study capture students’ awareness, perceptions, and experiences of institutional strategic planning, governance processes, resource allocation, digital infrastructure, organizational adaptation, and AI implementation rather than their direct knowledge of managerial or policy-level decision-making. As primary users and beneficiaries of university services, students’ perspectives provide valuable insights into the extent to which institutional initiatives are visible, accessible, and effectively implemented within the university environment.

In this regard, AI preparedness has become a crucial concept in current research on higher education. The degree to which organizations have the organizational culture, technology infrastructure, human capabilities, governance procedures, and strategic vision necessary for successful and long-lasting AI adoption is known as AI-ready (Chotia et al., 2025). Crucially, rather than being a static institutional state, AI readiness is increasingly seen as a dynamic organizational capability that changes via ongoing learning, adaptation, and strategic renewal. The Dynamic Capabilities Theory, which highlights an organization’s capacity to recognize opportunities and dangers, take strategic action to capture them, and reorganize internal resources in response to environmental change, forms the basis of this study (Sune and Gibb, 2026). According to this viewpoint, AI preparedness results from higher-order organizational capabilities that allow institutions to foresee, adjust, and change in response to technological disruption rather than just being a technological outcome.

Among these skills, one of the most important but little-studied factors influencing AI readiness is strategic foresight. The ability of a business to methodically investigate future developments, spot new technical trends, and create proactive plans in the face of uncertainty is referred to as strategic foresight (Biloslavo and Edgar, 2026). Higher education institutions can predict the effects of the rapid evolution of AI on pedagogy, governance, and institutional sustainability by using strategic foresight. However, actual research on how strategic foresight shapes AI preparation in higher education is limited, despite its theoretical significance.

In a similar vein, institutional adaptability has drawn more attention as a crucial organizational characteristic in settings that are changing quickly. According to Ramón and Koller (2026), it refers to an institution’s capacity to quickly detect changes in the environment, react appropriately, and reallocate resources in order to stay in line with new needs. In order to respond to technological disruption in higher education, students’ perceptions of institutional agility is crucial for modifying curricula, governance frameworks, and operational systems. However, there is still little empirical data regarding its mediating function in the development of AI preparedness. Three significant gaps are identified by a systematic evaluation of the literature. First, current research on AI in higher education ignores higher-order organizational qualities like institutional agility and strategic foresight in favor of concentrating on technology infrastructure, system adoption, or user approval. Second, previous research has tended to look at students’ perceptions of institutional agility, digital transformation capacity, and AI readiness separately, leading to a disjointed theoretical understanding. Third, there is a dearth of empirical data elucidating how internal organizational mechanisms, including digital transformation competence and institutional agility, translate strategic foresight into AI readiness. In the larger context of AI-enabled educational change, these gaps are very important. While a large portion of the literature focuses on institutional preparedness, contrasting research in educational psychology shows that individual-level characteristics like academic engagement, interest, and self-efficacy also have an impact on learning outcomes (Waqas et al., 2026). This implies that, especially in fields like mathematics education, where AI-driven adaptive learning systems are being used more frequently, successful AI integration in education necessitates alignment between institutional capabilities and learner-level psychological preparedness. To fill these gaps, this study develops and tests an integrated conceptual framework that looks at how strategic foresight influences AI readiness through the mediating roles of students’ perceptions of institutional agility and digital transformation capability in higher education institutions. By offering a more thorough and theoretically sound account of how organizational and strategic skills together determine institutional preparedness for AI adoption, the study adds to the expanding body of research on AI-driven transformation.

### Research objectives

1. To assess whether strategic foresight will positively predict AI readiness.
2. To assess whether strategic foresight will positively predict digital transformation capability.
3. To assess whether digital transformation capability will positively predict AI readiness.
4. To examine the mediating role of digital transformation capability on the relationship between strategic foresight and AI readiness.
5. To examine the mediating role of students’ perceptions of institutional agility on the relationship between strategic foresight and AI readiness.

### Contribution of the study

This study goes beyond explaining organizational readiness and digital transformation by showing how these institutional capabilities create an environment where people can effectively learn with and use AI. Rather than treating AI readiness as only a technological or organizational issue, the study highlights that strategic foresight, digital transformation capability, and students’ perceptions of institutional agility help universities build the conditions that support AI-enabled teaching, adaptive learning, and greater confidence in using AI technologies. In doing so, it contributes to understanding how organizational capabilities can encourage meaningful human learning and positive behavior change in AI-supported educational settings.

## Literature review

### Theoretical foundation of the study

This study is grounded in Dynamic Capabilities Theory, which explains how organizations develop the ability to respond to rapidly changing environments by sensing emerging opportunities, seizing those opportunities through effective resource utilization, and reconfiguring existing structures and processes to maintain competitiveness (Cavusgil and Deligonul, 2024). In this context, strategic foresight represents the sensing dimension of dynamic capabilities because it enables universities to identify emerging technological trends, anticipate future challenges, and recognize opportunities associated with digital transformation and artificial intelligence (AI). Through strategic foresight, institutions can develop an informed understanding of future demands and prepare appropriate responses before changes become disruptive.

Digital transformation capability reflects the seizing dimension of dynamic capabilities because it concerns the institution’s ability to mobilize resources, adopt digital technologies, and convert identified opportunities into practical initiatives. While strategic foresight allows universities to recognize the importance of AI and digital innovation, digital transformation capability enables them to allocate resources, develop technological infrastructure, and implement digital solutions that support institutional goals (Russo et al., 2025). Institutional agility, in contrast, represents the reconfiguration dimension because it reflects an institution’s ability to modify its structures, processes, and strategies in response to changing technological and environmental conditions. Agile institutions are better positioned to adjust existing practices, integrate new capabilities, and sustain transformation efforts over time (Naslund and Kale, 2020).

The inclusion of digital transformation capability and students’ perceptions of as mediators is theoretically justified because they represent different but complementary mechanisms through which strategic foresight influences AI readiness. Digital transformation capability focuses on the capacity to initiate and operationalize digital change through resource mobilization and technology implementation, whereas students’ perceptions of institutional agility emphasize the ability to continuously adapt and reorganize in response to new circumstances. Therefore, the two mediators are conceptually distinct, although closely related, and they operate as parallel mechanisms through which universities translate strategic awareness into AI preparedness. AI readiness is treated as an outcome rather than a dynamic capability because it represents the level of institutional preparedness achieved after capability-building processes have occurred. In other words, dynamic capabilities explain how universities develop and renew their abilities, while AI readiness reflects the resulting condition of being prepared to adopt and effectively use AI technologies (Jöhnk et al., 2021).

Furthermore, the use of student perceptions as indicators of these processes is theoretically appropriate because students are key stakeholders who directly experience the outcomes of institutional capabilities. Although students may not participate in high-level strategic decisions, their interactions with digital platforms, academic technologies, administrative systems, and institutional services provide meaningful insights into how strategic initiatives and technological changes are implemented and experienced at the operational level. Stakeholder theory suggests that the perceptions of affected groups provide valuable evidence regarding organizational practices and outcomes (Van Der Laan et al., 2008). Therefore, student responses in this study capture the observable manifestations and effectiveness of institutional capabilities rather than claiming that students possess expert knowledge of internal governance or managerial processes.

### Empirical review

#### Strategic foresight and AI readiness

Higher education institutions may anticipate future technical advancements, discern emerging trends, and establish proactive plans in the face of uncertainty thanks to strategic foresight, which is widely acknowledged as a crucial organizational competency. Foresight allows organizations to go from reactive adaptation to proactive digital transformation and strategic renewal in the light of the swift growth of artificial intelligence (AI) (Ibeh et al., 2024). AI integration is not just technological but also highly strategic, requiring long-term vision, leadership foresight, and institutional planning skills, according to recent empirical research in higher education and STEM education (Asghar et al., 2025). According to Vimbai and Notice (2024), AI readiness is defined as an institutional competency that indicates how well universities have the infrastructure, governance, human capital, and culture needed to successfully use AI technology. According to recent empirical research, AI-enabled learning environments that offer personalized learning pathways, scaffolded instruction, and adaptive feedback can greatly improve students’ engagement, conceptual understanding, and academic performance in mathematics (Junpeng, 2026). Recent research in mathematics education, in particular, shows that the incorporation of intelligent tutoring systems and generative AI tools promotes better problem-solving abilities and learner engagement, particularly when integrated within structured pedagogical frameworks (Liu et al., 2025). In this regard, Gqoli and Baidoo (2025) further stress that both cognitive and perceptual aspects, such as mathematics perception and the efficient use of AI tools in learning settings, have a substantial impact on student progress in mathematics. Their results demonstrate that, when paired with favorable learning attitudes and appropriate instructional design, AI-based instructional support enhances student achievements. When taken as a whole, these studies support the claim that AI in mathematics education is a pedagogical and structural shift that necessitates significant institutional preparedness and strategic alignment rather than just a technological advancement. Based on these discussions, the study proposed that;

*H1*: Strategic foresight will positively predict AI readiness.

#### Strategic foresight and digital transformation capability

The ability of an organization to incorporate digital technology into its procedures, organizational culture, and governance frameworks to accomplish strategic goals and boost performance is known as “digital transformation capability” (Gökalp and Martinez, 2022). This comprises data-driven decision-making systems, AI-supported teaching tools, and digital learning environments in higher education. By allowing organizations to foresee technological upheavals and strategically match digital investments with long-term institutional goals, strategic foresight improves digital transformation capability. Instead of implementing disjointed technological adoption attempts, institutions with greater foresight are more likely to create cohesive and sustainable digital transformation programs. AI-enabled learning environments can improve student engagement and academic performance, according to empirical data from studies on AI-supported mathematics education. This is especially true when these systems are integrated into sophisticated institutional digital infrastructures that support teaching and learning processes (Li et al., 2025). In addition, current research highlights that institutional capacity building, strategic planning, and the alignment of digital transformation initiatives with organizational objectives are all necessary for the successful implementation of artificial intelligence in higher education systems, especially in developing contexts (Khairullah et al., 2025). Because strategic foresight allows organizations to anticipate technological change, allocate resources efficiently, and align digital strategies with long-term institutional development objectives, these findings support the theoretical argument that strategic foresight is a critical antecedent of digital transformation capability in higher education institutions. Based on these discussions, the study hypothesized that;

*H2*: Strategic foresight will positively predict digital transformation capability.

#### Digital transformation capability and AI readiness

It is commonly acknowledged that a key factor in determining higher education institutions’ preparedness for AI is their capacity for digital transformation. AI technologies are more likely to be successfully adopted and scaled by institutions with sophisticated digital infrastructure, integrated information systems, and digitally savvy faculty. Digital transformation projects in mathematics education greatly improve instructional efficacy, especially through AI-assisted teaching tools and adaptive learning systems, according to recent studies by Wu et al. (2023). In a similar vein, Sun et al. (2025) stress that when AI integration in STEM education is backed by reliable digital systems, it enhances pedagogical quality and student-centered learning techniques. Digital transformation enhances AI preparedness from a capabilities standpoint by facilitating data accessibility, system interoperability, and organizational adaptability. As a result, institutional readiness for AI adoption is greatly aided by digital transformation capabilities. Dynamic Capabilities Theory serves as the foundation for this study’s conceptual framework (Cavusgil and Deligonul, 2024). It asserts that

- Institutions can foresee technological change thanks to strategic foresight.
- Technological and structural implementation is made possible by the capacity for digital transformation.
- Quick adaptation and resource reconfiguration are made possible by institutional agility.
- When taken as a whole, these skills define how prepared higher education institutions are for AI.

Based on these, the study hypothesized that;

*H3*: Digital transformation capability will positively predict AI readiness.

Based on hypotheses 1, 2, and 3, the study further proposed that;

*H4*: Digital transformation capability will mediate the relationship between strategic foresight and AI readiness.

*H5*: Students’ perceptions of Institutional agility will mediate the relationship between strategic foresight and AI readiness.

Figure 1 illustrates the conceptual framework of the study. From Figure 1, SFO represents the independent variable, DTC and SPIAG serves as the mediating variables, while AI_R also serve as the dependent variable. Furthermore, SFO show a direct influence on DTC, SPIAG, and AI_R. in addition DTC and SPIAG shows a direct influence on AI_R. Finally, DTC and SPIAG mediate the relationship between SFO and AI_R.

**Figure 1:**
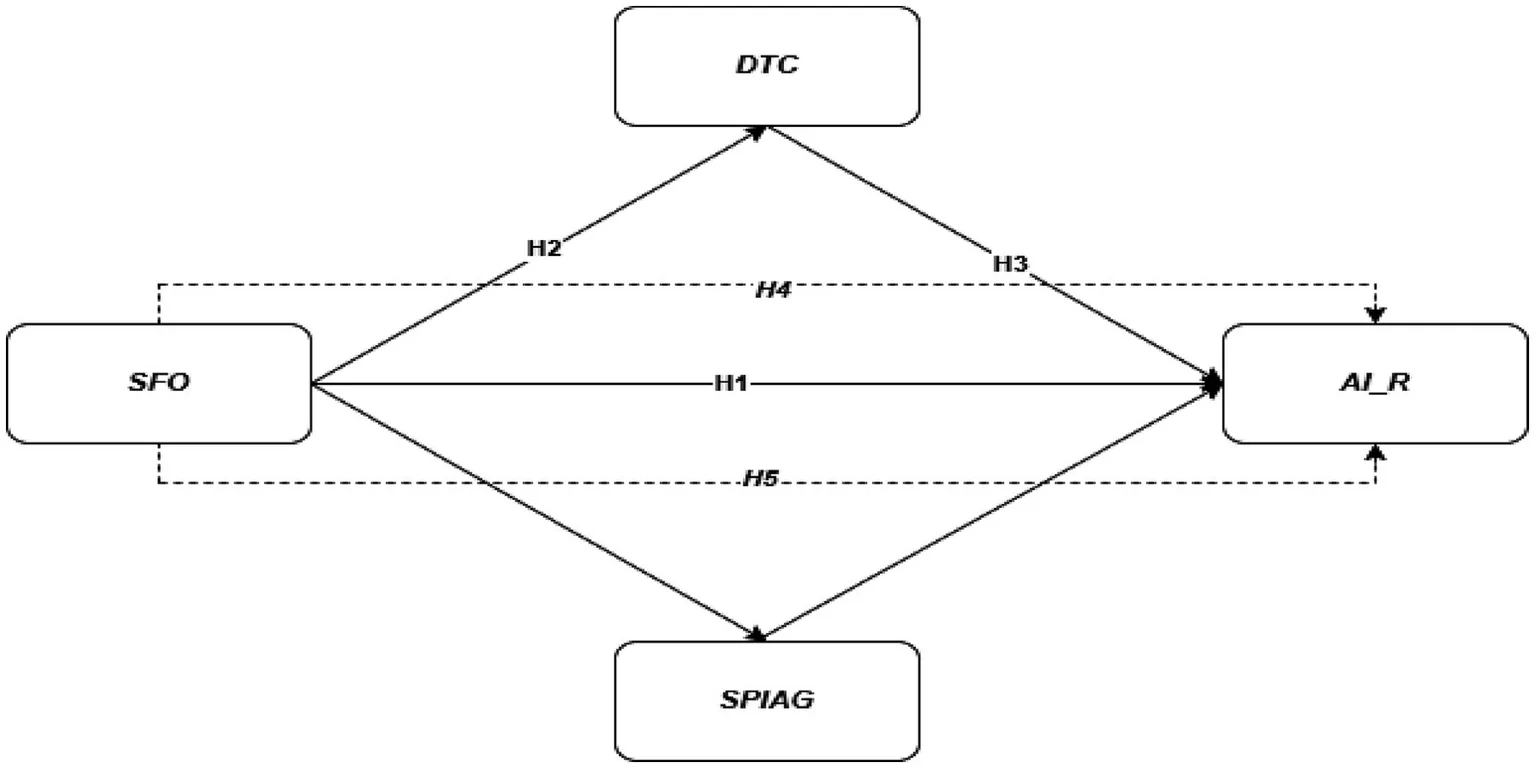
Conceptual framework (Source: Author Creation, 2026).

## Methodology

### Design

The study used a cross-sectional SEM analysis research design to examine the relationships among strategic foresight, AI readiness, digital transformation capability, and institutional agility. This design was chosen because it allows the researcher to describe the current state of these variables as they exist in real settings, while also testing how they are related to one another. It is also appropriate for this study because none of the variables were manipulated. Instead, the focus was on observing and analyzing naturally occurring conditions within institutions to understand how these factors interact.

### Population, sample size, and sampling techniques

The study population comprised university students at the University of Skills Training and Entrepreneurial Development (USTED), Kumasi. The university was selected because the use of AI in academic learning is allowed by students, irrespective of their level of education at the university. The study precisely targeted students pursuing Mathematics Education, Information Technology Education, and Construction Education. These programs were selected because the corresponding author was assigned to teach algebra, engineering math II, and Discrete Mathematics at various levels of education. The selected student was in their level 100 s and level 200 s. The study sample size was determined based on the complete online questionnaire completed by the selected students. Based on the data extracted from the Google Form, 311 students were able to complete the online questionnaire completely. The sample size used in this study was considered adequate for conducting quantitative analyses, particularly regression and mediation analyses. A sample of this magnitude provides sufficient statistical power and allows for the reliable examination of relationships among the study variables (Hair et al., 2014).

Moreover, Table 1 summarizes the demographic characteristics of the 311 students who participated in the study. The respondents were mainly from the BSc. Mathematics Education program (59.8%), followed by students from the BSc. Information Technology Education program (33.1%), while a small percentage (7.1%) were from other programs. In terms of academic level, the participants were relatively evenly distributed, with 55.9% being second-year students and 44.1% being first-year students. Most respondents were within the age range of 21–23 years (40.5%), followed by those aged 18–20 years (33.4%), while fewer students were in the older age categories. The gender distribution showed that male students represented a slightly higher proportion (53.1%) than female students (45.9%), with only 1.0% choosing not to disclose their gender. Furthermore, the majority of respondents (78.5%) had previous experience using AI tools, indicating a considerable level of familiarity with AI technologies. Similarly, most students (65.9%) reported having 2 years of experience with digital learning systems, while 34.1% had 1 year of experience. In all, the demographic profile suggests that the participants had relevant exposure to digital technologies and learning environments, making them suitable for providing informed perceptions related to the study objectives.

**Table 1:** Demographics characteristics of respondents.

Demographic variable	Category	Frequency (*n*)	Percentage (%)
Program	BSc. Mathematics Edu.	186	59.8
	BSc. Information Technology Edu.	103	33.1
	Other	22	7.1
Year of study	1 year	137	44.1
	2 year	174	55.9
Age	18–20 years	104	33.4
	21–23 years	126	40.5
	24–26 years	57	18.3
	27 years and above	24	7.8
Gender	Male	165	53.1
	Female	143	45.9
	Prefer not to say	3	1.0
Prior AI use	Yes	244	78.5
	No	67	21.5
Experience with digital learning systems	1 year	106	34.1
	2 years	205	65.9

Moreover, a single-institution convenience sample was employed to recruit participants. This method involved selecting respondents who were easily accessible and willing to participate in the study. The choice of this sampling approach was influenced by the ease of reaching university students and the use of an online questionnaire as the primary data collection instrument. Furthermore, the questionnaire was distributed electronically through student groups, institutional communication platforms, and social media networks associated with the participating universities. Before completing the survey, participants were informed that their responses would remain confidential and anonymous. They were also assured that the information collected would be used exclusively for academic research purposes. Participation was entirely voluntary, and respondents were free to decide whether to take part in the study.

### Research instrument and measures

The research instrument for the study data collection was a structured questionnaire, which was designed using Google Forms based on the study constructs. The measurement items for strategic foresight, AI readiness, digital transformation capability, and students’ perceptions of institutional agility were all designed by the author. Each construct comprised 10 measurement items or statements. The items were all measured using a five-point Likert Scale ranging from 1 (strongly disagree) to 5 (strongly agree). Before the main data collection, a pilot test was carried out using 30 students from the study area to check the reliability of the questionnaire. The results showed very high reliability values for all the constructs: strategic foresight (0.968), AI readiness (0.968), digital transformation capability (0.960), and students’ perceptions of institutional agility (0.924). All these values were above the recommended minimum of 0.70 suggested by McCrae et al. (2011), indicating that the items were consistent and dependable.

An initial pool of 40 items was generated following a comprehensive review of previous studies and theoretical literature relevant to each construct. Each item was designed to capture a distinct aspect of the underlying concept while ensuring relevance to the institutional context addressed in this study. To establish content validity, the initial questionnaire was reviewed by three experts with expertise in artificial intelligence. The experts evaluated each item for clarity, relevance, representativeness, and appropriateness using established content-validation criteria. Their comments resulted in revisions to wording, elimination of redundant items, and refinement of ambiguous statements. The revised questionnaire was subsequently pretested through cognitive interviews and/or a pilot study involving 30 participants representative of the target population. Participants were asked to comment on the clarity, interpretation, and comprehensibility of the items, leading to minor modifications in wording and item sequencing. Where applicable, the questionnaire was translated using a forward-backward translation procedure to ensure semantic equivalence between language versions. Following expert review and pilot testing, the questionnaire was refined from an initial pool of 40 items to a final instrument comprising 311, thereby enabling evaluation of the instrument’s content validity and construct coverage.

### Data collection procedure

Data were collected using an online questionnaire administered through a survey platform. Before the data collection began, permission was obtained from the relevant university authorities to ensure that all ethical requirements were met. The purpose of the study was clearly explained to the participants, and they were asked to give their informed consent before taking part. Participation was completely voluntary, and respondents were assured that their responses would remain anonymous and confidential. The questionnaire link was shared with selected students through appropriate online channels. Participants were given enough time to complete the survey at their own pace so that they could provide thoughtful and accurate responses.

### Procedure for data analysis

The data were analyzed using IBM SPSS Statistics and IBM SPSS Amos (ver. 23). First, descriptive statistics, mainly frequencies, were used to check the dataset and identify any missing responses. This step helped ensure that the data were complete and suitable for further analysis. Next, Exploratory Factor Analysis (EFA) was carried out using SPSS to explore the underlying structure of the measurement items and to see how well they grouped under their expected constructs. Items with factor loadings of at least 0.50 were retained, following the guideline suggested by Marsh et al. (2020). After that, Confirmatory Factor Analysis (CFA) was conducted in Amos to confirm the measurement model obtained from the EFA. The model fit was evaluated using standard indices such as CFI, TLI, RMSEA, and the Chi-square/degree of freedom ratio. These measures were used because they give a clear indication of how well the proposed model fits the observed data, in line with Hair et al. (2014). Furthermore, discriminant validity was also assessed to make sure that the constructs were distinct from each other and that multicollinearity was not a problem. This helped confirm that each construct measured a different aspect of the study. Furthermore, the structural model was tested to examined the individual direct effects. Finally, the mediating effects were examined using the bootstrapping procedure with 5,000 resamples and a 95% confidence interval, as this approach provides a more reliable assessment of indirect effects in structural models. Following established recommendations, the significance of the mediation relationships was determined by assessing the bootstrap confidence intervals of the indirect effects. A mediation effect was considered statistically significant when the confidence interval did not contain zero. Accordingly, the analysis assessed whether digital transformation capability and students’ perceptions of institutional agility serve as mediating mechanisms through which strategic foresight influences AI readiness.

### Assessment of common method Bias

The issue of common method variance (CMV) was considered in this study because all the data were collected from the same group of respondents at the University of Skills Training and Entrepreneurial Development (USTED), Kumasi campus. Podsakoff and Organ (1986) explain that CMV can affect the validity of results in survey research, so it is important to check for it. To test for this, Harman’s single-factor approach was used through exploratory factor analysis (EFA). The results showed that five factors had eigenvalues greater than 1 and together explained 71.088% of the total variance. The first factor accounted for only 20.774% of the variance, which suggests that common method bias was not a major concern in this study. Additional checks also showed that the data were suitable for analysis. The Kaiser-Meyer-Olkin (KMO) value was 0.956, which indicates excellent sampling adequacy (Tabachnick and Fidell, 2007). Bartlett’s test of sphericity was also significant (*χ*^2^ = 5718.859, df = 300, *p* < 0.001), confirming that the variables were sufficiently related for factor analysis. In all, although the sample size was 311, all the necessary statistical checks were met, showing that the data were reliable and appropriate for testing the study’s hypotheses.

### Normality analysis

Table 2 presents the results of the Kolmogorov–Smirnov and Shapiro–Wilk tests, which were conducted to assess whether the data from the four class groups were normally distributed. Assessing normality is an important step before selecting the most appropriate statistical techniques for data analysis. The Kolmogorov–Smirnov test produced statistics of 0.078 for IAG, 0.130 for SF, 0.089 for DTC, and 0.097 for AI_R, based on a sample size of 311. The Shapiro–Wilk test yielded values of 0.962 (*p* = 0.069) for IAG, 0.959 (*p* = 0.071) for SF, 0.963 (*p* = 0.087) for DTC, and 0.952 (*p* = 0.097) for AI_R. Since all the Shapiro–Wilk *p*-values were greater than the recommended threshold of 0.05, the results suggest that the data did not significantly deviate from a normal distribution. Therefore, the assumption of normality was satisfied, indicating that parametric statistical methods could be appropriately applied in the subsequent analyses.

**Table 2:** Tests of normality.

Constructs	Kolmogorov–Smirnov^a^			Shapiro–Wilk		
	Statistic	df	Sig.	Statistic	df	Sig.
SPIAG	0.078	311	0.075	0.962	311	0.069
SF	0.130	311	0.054	0.959	311	0.071
DTC	0.089	311	0.061	0.963	311	0.087
AI_R	0.097	311	0.074	0.952	311	0.097

^a^Lilliefors Significance Correction, NB: IAG, Institutional Agility; SF, Strategic Foresight; DTC, Digital Transformation Capability; AI_R, AI Readiness.

The results indicate that the data satisfied the normality assumption required for multivariate analysis. The *p*-values from both the Kolmogorov–Smirnov and Shapiro–Wilk tests for all variables were greater than the recommended threshold of 0.05, indicating that the data did not deviate significantly from a normal distribution. The Kolmogorov–Smirnov results were interpreted using the Lilliefors significance correction, which accounts for situations where population parameters are estimated from the sample. Since the assumption of normality was met, the data were considered suitable for multivariate statistical techniques, including correlation, regression, and structural equation modeling (SEM).

Figure 2 shows how respondents scored across four variables: SFO, SF, AI_R, DTC, and SPIAG. In general, responses are fairly similar across all four areas, with most scores clustering toward the higher end of the scale. SFO has a slightly higher median than the others, indicating that respondents rated it a bit more positively. The spread of responses is also comparable across the variables, showing that people answered in a fairly consistent way. However, a few low values appear in each variable, indicating that a small number of respondents gave lower ratings than the majority. In all, the pattern suggests that responses are generally positive and fairly consistent across all four variables.

**Figure 2:**
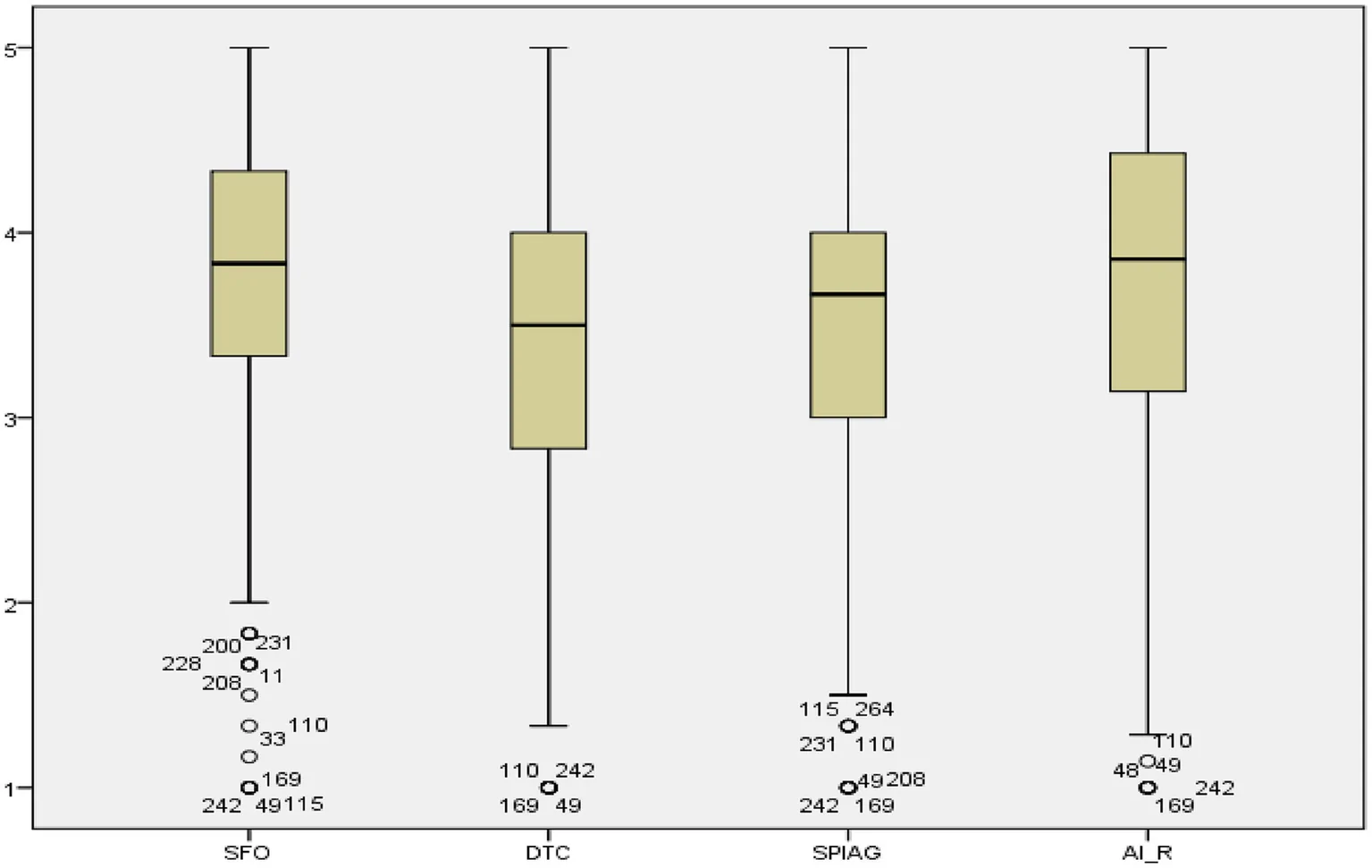
Box plot (Source: SPSS output).

### Assessment of validity and reliability

To ensure that the survey items truly reflected the concepts they were designed to measure, an Exploratory Factor Analysis (EFA) was carried out using SPSS (ver. 23). EFA is a method used to explore how different items group together and reveal the underlying patterns in the data, especially when those patterns are not directly visible. In simple terms, it helps confirm whether related questions are actually measuring the same idea (Zakariya and Massimiliano, 2021). The analysis focused on four key areas of the study: strategic foresight, AI readiness, digital transformation capability, and institutional agility. Each of these areas was measured using several questionnaire items. Students’ perceptions of Institutional agility and digital transformation capability each had five items, AI readiness had six items, and strategic foresight had four items, as shown in Table 3.

**Table 3:** Exploratory factor analysis (EFA), KMO, and Bartlett’s test.

Rotated component matrix				
Measurement items	Component			
	1	2	3	4
SFO1			0.719	
SFO3			0.683	
SFO4			0.763	
SFO5			0.730	
SFO6			0.718	
SFO10			0.657	
DTC2		0.718		
DTC4		0.768		
DTC5		0.756		
DTC6		0.772		
DTC7		0.721		
DTC10		0.652		
SPIAG2				0.668
SPIAG3				0.678
SPIAG6				0.732
SPIAG7				0.717
SPIAG8				0.720
SPIAG9				0.708
AI_R1	0.762			
AI_R2	0.836			
AI_R3	0.789			
AI_R4	0.799			
AI_R6	0.711			
AI_R7	0.699			
AI_R10	0.704			

KMO and Bartlett’s test		
TVE		71.088%
Kaiser-Meyer-Olkin Measure of Sampling Adequacy.		0.956
Bartlett’s Test of Sphericity	Approx. Chi-Square	5718.859
	df	300
	Sig.	0.000
Determinant		1.78E-09

During the process, any item that did not strongly relate to its intended group, specifically those with factor loadings below 0.50, was removed to improve the overall quality of the measurement (Kosteniuk et al., 2017; Watkins, 2018). After this refinement, the remaining items clearly aligned with their respective constructs: five items each for students’ perceptions of institutional agility and digital transformation capability, six items for AI readiness, and four items for strategic foresight. Overall, the results showed that the model explained 71.088% of the total variation in the data, which is above the commonly accepted minimum of 50%, indicating a good level of explanatory power. In addition, the Kaiser–Meyer–Olkin (KMO) value was 0.956, which is well above the recommended threshold of 0.60. This confirms that the data were highly suitable for factor analysis (Marsh et al., 2020).

Bartlett’s test of sphericity, computed from the initial correlation matrix, was statistically significant (*χ*^2^ = 5718.859; *p* < 0.001), indicating that the variables were sufficiently interrelated and therefore appropriate for Exploratory Factor Analysis (EFA) (de Winter et al., 2009). In addition, the determinant of the correlation matrix was examined to assess potential multicollinearity issues. According to de Winter et al. (2009), the determinant should be greater than zero to confirm that the matrix is non-singular and suitable for factor analysis. In this study, the determinant value was 8.03E-06, which, although very small, remained above zero and thus satisfied this assumption.

Following this, Confirmatory Factor Analysis (CFA) was conducted using AMOS (Ver. 23) to validate the factor structure derived from the EFA. CFA is used to evaluate how well the observed data fit a proposed measurement model and to confirm the relationships between observed indicators and their underlying latent constructs (Akomah and Ramani, 2023). The CFA results are presented in Table 4 and Figure 3. The standardized factor loadings are expected to exceed the recommended threshold of 0.50, indicating that each item contributes meaningfully to its respective construct. Finally, the reliability of the measurement model was assessed using Cronbach’s Alpha and McDonald’s Omega. The results indicated that all constructs exceeded the recommended threshold of 0.70 (Marsh et al., 2020; Rönkkö and Cho, 2022), demonstrating strong internal consistency among the measurement items.

**Table 4:** Confirmatory factor analysis (CFA).

Constructs/measurement items	Std. estimate
AI_R: Cronbach’s Alpha ( α ) = 0.939; McDonald’s Omega ( Ω ) = 0.894	
AI_R1	0.831
AI_R2	0.861
AI_R3	0.807
AI_R4	0.859
AI_R6	0.842
AI_R7	0.778
AI_R10	0.838
DTC: Cronbach’s Alpha ( α ) = 0.918; McDonald’s Omega ( Ω ) = 0.800	
DTC2	0.799
DTC4	0.828
DTC5	0.820
DTC6	0.762
DTC7	0.833
DTC10	0.803
SFO: Cronbach’s Alpha ( α ) = 0.872: McDonald’s Omega ( Ω ) = 0.849	
SFO1	0.752
SFO3	0.790
SFO4	0.723
SFO5	0.839
SFO6	0.777
SFO10	0.693
SPIAG: Cronbach’s Alpha ( α ) = 0.916; McDonald’s Omega ( Ω ) = 0.870	
SPIAG2	0.708
SPIAG3	0.757
SPIAG6	0.832
SPIAG7	0.820
SPIAG8	0.855
SPIAG9	0.818

**Figure 3:**
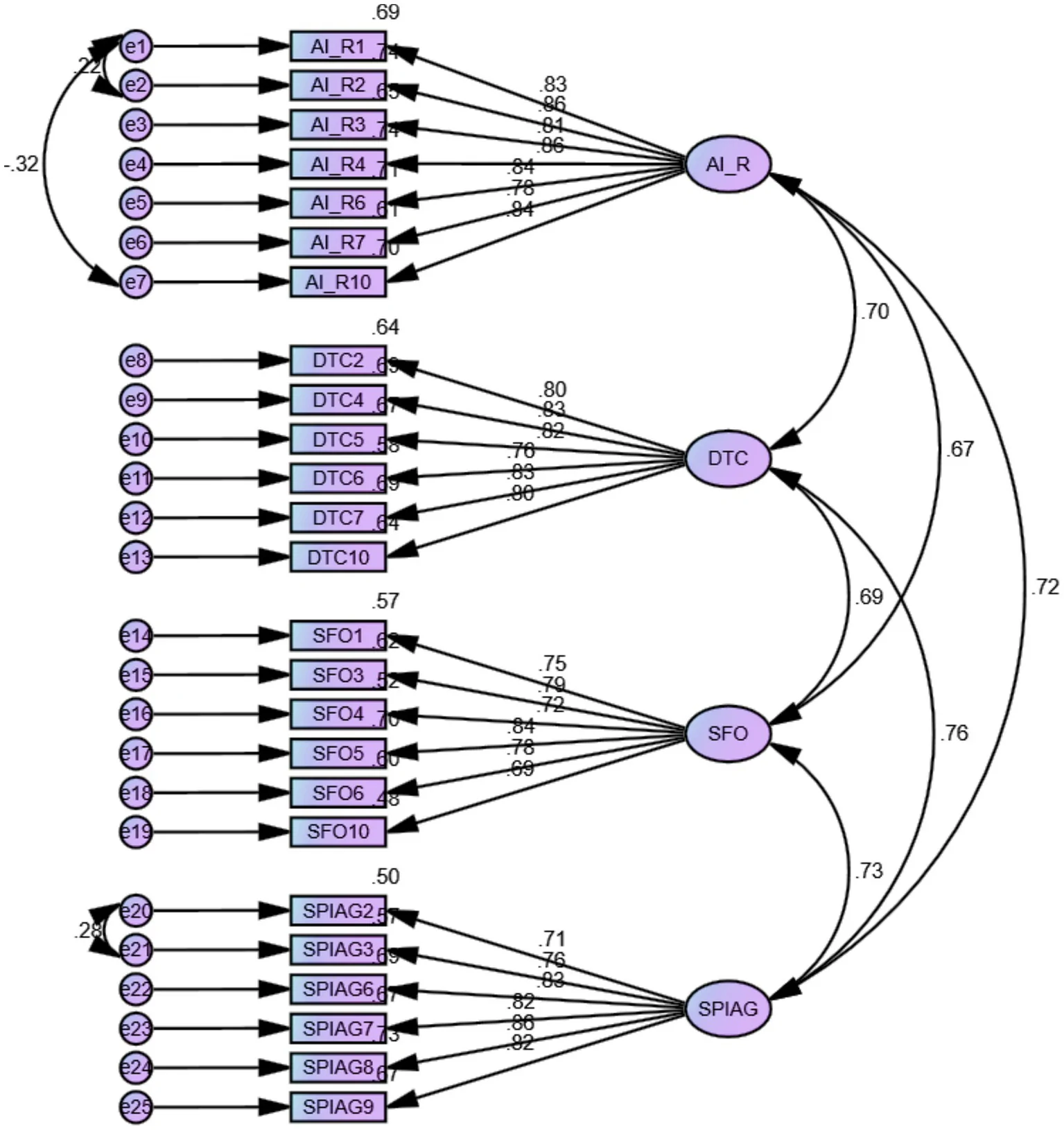
Confirmatory factor analysis (CFA) (Source: Author creation, 2026).

According to Fornell and Larcker (1981), convergent validity necessitates composite reliability (CR) of at least 0.7 rather than a minimum Average Variance Extracted (AVE) of 0.5, and each construct successfully attained all of these criteria. Figure 3 shows the illustration for the confirmatory factor analysis.

Figure 3 presents the graphical representation of the measurement model shown in Table 2. It indicates that AI_R and SPAIG are each measured using five items, while DTC and SFO are each measured using four items. The figure also shows the correlations among the four constructs (AI_R, SPAIG, DTC, and SFO), illustrating how they are related within the proposed model. To achieve a better-fitting measurement model, correlations were added between the error terms e1↔e2, e1↔e7, and e20↔e21. These modifications were guided by the modification indices and are theoretically acceptable because the items involved are likely to share some common variance due to similarities in their wording or measurement characteristics. As a result, the overall model fit was improved while preserving the theoretical integrity of the model.

#### Model fit indices

The structural equation model fits the observed data well, as shown by the fit indices in Table 5. The Chi-square value (CMIN = *493.326*) with 266 degrees of freedom results in a normed Chi-square (CMIN/DF = *1.855*), which falls within the commonly accepted range of 1 to 3. This suggests that the model represents the data well (Hayduk and Littvay, 2012). The incremental fit indices also point to a strong model fit. The Tucker-Lewis Index (TLI = 0.954) indicates an excellent fit, while the Comparative Fit Index (CFI = 0.959) is above the recommended threshold of 0.95. Together, these values show that the model explains the data very well and performs strongly in comparison with a null model (Bagozzi et al., 1991; Ding et al., 1995). The absolute fit measures tell the same story. The Goodness-of-Fit Index (GFI = 0.955) is above the acceptable benchmark of 0.90, suggesting a good level of fit. The Standardized Root Mean Square Residual (SRMR = 0.050) is also well below 0.08, meaning there is only a small difference between the observed and predicted values (Marsh et al., 2020; Sarmento and Costa, 2019; Shek and Yu, 2014). Finally, the Root Mean Square Error of Approximation (RMSEA = 0.054) falls within the acceptable range, indicating a reasonable level of approximation error. Although it is slightly above the very strict cutoff of 0.06, it still supports the conclusion that the model fits the data well overall.

**Table 5:** Model fit measures.

Measures	Estimate	Standard	Interpretation	Source (citation)
CMIN	493.326	–	–	–
DF	266	–	–	–
CMIN/DF	1.855	Between 1 and 3	Excellent	Hayduk and Littvay (2012)
CFI	0.959	>0.95	Excellent	Bagozzi et al. (1991)
TLI	0.954	>0.90	Good fit	Ding et al. (1995)
GFI	0.955	>0.8 Acceptable; >0.9 good fit	Good	Marsh et al. (2020)
SRMR	0.050	<0.08	Excellent	Shek and Yu (2014)
RMSEA	0.054	<0.060	Excellent	Bagozzi et al. (1991)
NFI	0.956	>0.95	Excellent	Hurley et al. (2017)
PClose	0.184	>0.05	Excellent	Marsh et al. (2020)

The PClose value of 0.184 is above the 0.05 threshold, meaning the RMSEA is not significantly different from what we would expect in a well-fitting model. This is consistent with the findings of Marsh et al. (2020). In the same way, the Normed Fit Index (NFI = 0.956) is slightly above the recommended cut-off point of 0.95, which points to a very good model fit, as also noted by Hurley et al. (2017). Taken together, these results suggest that the proposed model fits the data well and is both statistically reliable and theoretically meaningful.

To further check whether the constructs are truly different from one another, the study compared the square root of the Average Variance Extracted (√AVE) with the correlations between the constructs. The basic idea is that each construct should relate more strongly to its own indicators than to other constructs. In other words, the √AVE should be higher than the inter-construct correlations (dos Santos and Cirillo, 2023). From Table 6, the lowest √AVE value is 0.764, and this is still higher than the highest correlation value of 0.759. This clearly shows that the constructs are well separated from each other, confirming good discriminant validity.

**Table 6:** Discriminant validity.

Constructs	CR	AVE	AI_R	SDTC	SFO	SPIAG
AI_R	0.940	0.691	**0.831**			
DTC	0.918	0.652	0.696^***^	**0.808**		
SFO	0.893	0.584	0.669^***^	0.687^***^	**0.764**	
SPIAG	0.914	0.6400	0.722^***^	0.759^***^	0.726^***^	**0.800**

The bolded values are the roots of AVEs. ***Significance.

Another important issue in model estimation is multicollinearity, which happens when predictor variables are strongly related to each other. In most cases, correlations around 0.50 or higher can be seen as fairly strong and may suggest some overlap between variables. In this study, the highest correlation recorded was 0.710, but this was still not high enough to cause serious problems or affect the reliability of the model. The CFA results also showed that the data fit well for further analysis. In all, this means the dataset was appropriate for testing the study’s hypotheses.

## Research results

Structural Equation Modelling (SEM) was applied to estimate the path relationships using Amos (ver. 23), and the results are presented in Table 7 and Figure 4. To test the mediation effects, a bootstrapping approach with 5,000 resamples and 95% bias-corrected confidence intervals was employed.

**Table 7:** Paths estimates.

Direct effect	Std. estimate	S.E.	C.R.	*p*-value
SFO → DTC	0.727	0.078	9.321	<0.05
DTC → AI_R	0.291	0.080	3.638	<0.05
SFO → AI_R	0.217	0.103	2.107	<0.05

Indirect effect	Std. estimate	Lower B.C.	Upper B.C.	*p*-value
SFO → DTC → AI_R	0.249	0.115	0.412	<0.05
SFO → SPIAG → AI_R	0.221	0.079	0.390	<0.05

**Figure 4:**
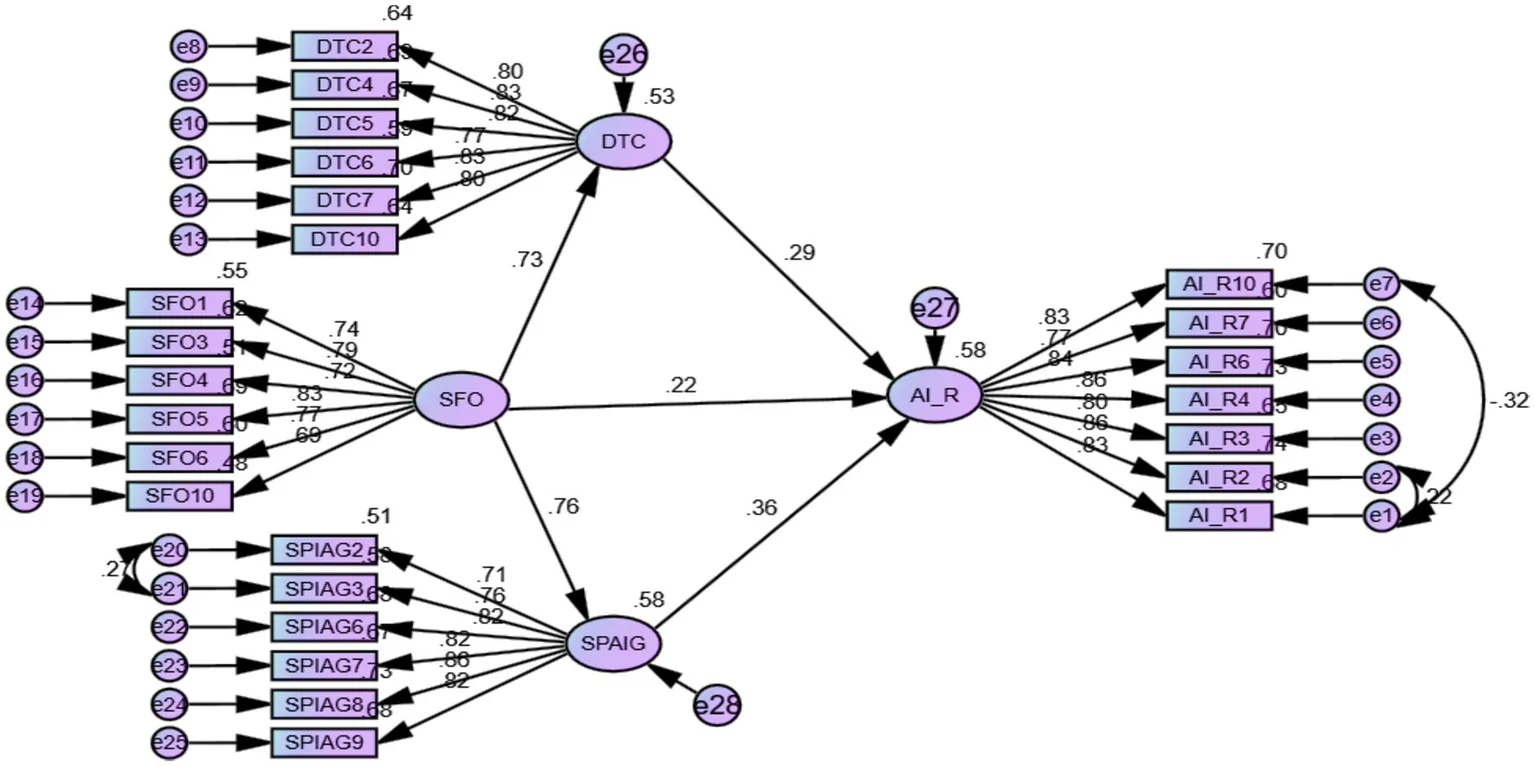
Path estimate illustrates (Source: Author Creation, 2026).

To start with, *H1* proposed whether SFO will positively predict AI_R. The results in Table 7 show that SFO positively predict AI_R, so *H1* is supported. In addition, *H2* suggested whether SFO will positively predict DTC. This was confirmed in the analysis, as SFO positively predict DTC, leading to the acceptance of *H2*. Moreover, *H3* examined whether DTC will positively predict AI_R, and the findings again showed that DTC positively predict AI_R. Therefore, *H3* is also supported. Furthermore, for *H4*, the study looked at whether DTC will mediate the relationship between SFO and AI_R. The results show that SFO positively predict DTC, and DTC in turn predict AI_R. Even though SFO positively predict AI_R, the presence of DTC reduces this effect, meaning the mediation is partial. A bootstrapping test with 5,000 samples confirmed that the indirect effect (0.289) is statistically significant, since the confidence interval (0.180 to 0.441) does not include zero. Based on this, *H4* is accepted. Finally, *H5* tested whether SPIAG mediates the relationship between SFO and AI_R. The results show a similar pattern: SFO positively predict IAG, and IAG positively predict AI_R. SFO positively predict AI_R, so this is also a partial mediation. The bootstrapped indirect effect (0.291) was significant, with a confidence interval (0.175 to 0.445) that excludes zero. Therefore, *H5* is also supported.

## Discussion of results

To start, the finding that strategic foresight positively predict AI readiness suggests that organizations that actively anticipate future technological trends are better prepared to adopt and implement artificial intelligence systems. Strategic foresight enhances environmental scanning, long-term planning, and proactive decision-making, which collectively improve an organization’s preparedness for emerging technologies such as AI. This result is consistent with the work of Alqershi et al. (2026), who emphasize that strategic foresight strengthens organizational sensing capabilities and improves preparedness for technological disruptions, including digital and AI-related transformations. Similarly, a recent study by Mohammad et al. (2025) found that strategic foresight significantly improves digital readiness by enabling organizations to align resources, skills, and strategies toward future technological demands. This suggests that AI readiness is not only a technical issue but also a strategic capability shaped by foresight practices.

Moreover, the result, indicated that strategic foresight positively predict digital transformation capability, implies that organizations with stronger foresight practices are more capable of building and sustaining digital transformation processes. This finding aligns with the study of Ellstr et al. (2026), who argue that strategic foresight enhances dynamic capabilities and supports organizational transformation in digital contexts. In addition, studies such as those by Bahyan et al. (2026), confirm that foresight improves digital transformation capability by fostering adaptability, innovation, and strategic alignment with emerging technologies. Therefore, organizations that invest in foresight are more likely to succeed in digital transformation initiatives.

In addition, the finding that digital transformation capability significantly influences AI readiness suggests that organizations with strong digital capabilities are more prepared to integrate artificial intelligence into their operations. Digital transformation capability provides the technological infrastructure, data integration systems, and organizational agility required for AI adoption. This result is supported by Shatila (2025), who explain that digital transformation capability enhances organizational agility and readiness for advanced technologies such as AI by enabling continuous adaptation and innovation. Similarly, recent empirical evidence by Kristiani (2025) highlights that digital transformation maturity is a key determinant of AI readiness because it strengthens data-driven decision-making and technological integration capacity. Thus, organizations with higher digital transformation capability are more likely to achieve successful AI readiness.

Furthermore, the finding that digital transformation capability (DTC) mediates the relationship between strategic foresight orientation (SFO) and artificial intelligence readiness (AI_R) suggests that strategic foresight alone is not sufficient to enhance AI readiness unless higher educational institutions are able to convert that foresight into practical digital capabilities. This finding aligned with the study of Weritz et al. (2025) who argue that digital capabilities act as the bridge between strategic orientation and successful digital outcomes. However, what is less explored in existing literature is how strategic foresight specifically translates into AI readiness through DTC in higher educational institutions. This study, therefore, fills that gap by showing that DTC is the critical pathway through which foresight becomes actionable AI preparedness.

Similarly, the result that students’ perceptions of institutional agility mediates the relationship between strategic foresight and AI readiness highlights the importance of flexibility in organizational systems for converting long-term strategic thinking into technological readiness. Even when higher educational institutions possess strong foresight about future AI-driven changes, their ability to respond effectively depends on how quickly and flexibly their institutional structures can adapt. Students’ perceptions of Institutional agility allows higher educational institutions to adjust policies, processes, and decision-making structures in response to emerging technological opportunities, thereby enabling AI readiness. This is consistent with studies which describes agility as a dynamic capability that supports rapid adaptation to environmental and technological change (Ajgaonkar et al., 2026; Baškarada and Koronios, 2026). Similarly, Gong and Ribiere (2026) emphasize that organizational agility strengthens the translation of strategic intentions into digital transformation outcomes. However, most existing studies have not examined students’ perceptions of institutional agility as a mediating mechanism between strategic foresight and AI readiness, especially within higher educational contexts. This study, therefore, extends the literature by demonstrating that students’ perceptions of institutional agility is a necessary bridge that converts foresight into real AI preparedness.

### Practical, educational, and theoretical implications

To start with, the findings of this study offer several practical insights for organizational leaders, policymakers, and practitioners seeking to strengthen AI readiness. The positive relationship between strategic foresight and AI readiness suggests that organizations should adopt a more forward-looking approach to strategic planning. This involves continuously monitoring changes in the external environment, anticipating technological developments, and incorporating future-oriented thinking into strategic decision-making. Such practices enable organizations to identify opportunities and potential disruptions early, allowing them to prepare more effectively for AI implementation. The study further shows that digital transformation capability plays a central role in translating strategic foresight into AI readiness. This implies that organizations should complement their long-term strategic vision with sustained investments in digital infrastructure, data management systems, employee digital competencies, and technology-enabled business processes. Also, the mediating roles of digital transformation capability and students’ perceptions of institutional agility indicate that AI readiness depends not only on strategic planning but also on an organization’s ability to adapt and respond to changing technological demands. Managers should therefore foster agile organizational structures, encourage innovation, streamline decision-making processes, and promote a culture of continuous learning to ensure that strategic intentions are effectively converted into AI adoption and utilization.

Moreover, the results also have important implications for higher education institutions and professional training providers. As organizations increasingly rely on AI technologies to improve performance and competitiveness, there is a growing need to prepare graduates and professionals with the knowledge and skills required to navigate digital transformation. The findings suggest that strategic foresight should become an integral component of business, management, and information systems curricula. Students should be equipped with the ability to anticipate future trends, evaluate emerging technologies, and make strategic decisions in uncertain environments. At the same time, educational institutions should provide practical learning experiences that enhance digital competencies, including data analytics, digital innovation, AI applications, and technology management. Leadership development programs should also emphasize students’ perceptions of institutional agility by equipping future managers with skills in adaptive leadership, change management, collaboration, and organizational resilience. Such educational initiatives will produce graduates who are not only technically competent but also capable of leading organizations through complex digital and AI-driven transformations.

Finally, this study contributes to the growing literature on strategic foresight, digital transformation, and AI readiness by providing empirical evidence on the mechanisms through which these constructs are connected. While previous studies have generally examined these concepts independently, this research demonstrates that strategic foresight contributes to AI readiness both directly and indirectly through digital transformation capability and institutional agility. These findings deepen our understanding of how organizations convert future-oriented strategic thinking into practical readiness for AI adoption. The study further highlights that developing digital capabilities alone may not be sufficient; organizations must also possess the agility needed to respond quickly to changing technological and environmental conditions. By integrating these relationships into a single framework, the study provides a more comprehensive explanation of the organizational capabilities that underpin AI readiness. This integrated perspective enriches existing theory by illustrating that AI readiness is the outcome of a combination of strategic vision, digital capability development, and organizational adaptability rather than any single organizational resource or capability.

## Conclusion

The study was based on five research hypotheses, which were *H1:* “Strategic foresight will positively predict AI readiness,” *H2:* “Strategic foresight positively predict digital transformation capability,” *H3:* “Digital transformation capability positively predict AI readiness,” *H4:* “Digital transformation capability will mediate the relationship between strategic foresight and AI readiness,” and *H5:* Students’ perceptions of institutional agility will mediate the relationship between strategic foresight and AI readiness.” The study concluded that strategic foresight positively predicted AI readiness. Moreover, the study established that strategic foresight positively predicted digital transformation capability. In addition, the study demonstrated that digital transformation capability positively predicted AI readiness. Furthermore, the study indicated that digital transformation capability mediates the relationship between strategic foresight and AI readiness. Finally, the evidence points to the conclusion that students’ perceptions of institutional agility mediate the relationship between strategic foresight and AI readiness.

## Limitations and future research directions

This study has several limitations that should be considered when interpreting the findings. First, the use of a cross-sectional research design means that the relationships identified among strategic foresight, digital transformation capability, institutional agility, and AI readiness were examined at a single point in time. Therefore, the study cannot fully establish causal relationships among the constructs. Future research could adopt longitudinal approaches to examine how these capabilities develop over time and how they influence organizations’ readiness for artificial intelligence adoption. Second, the study relied on data collected from a single source, which may increase the possibility of common-method variance. Although efforts were made to minimize this concern, future studies could strengthen the reliability of findings by collecting data from multiple sources, such as managers, employees, and institutional records.

The findings should also be interpreted within the context of the sampling approach and research setting. The use of convenience sampling and data collected from a single institution may limit the generalizability of the results to other organizational contexts. Future studies should consider larger and more diverse samples across multiple institutions, industries, and geographical locations to determine whether the observed relationships remain consistent in different settings. Additionally, the possibility of respondent-construct mismatch should be acknowledged, as respondents may not always have complete knowledge of all the organizational-level capabilities examined in this study. Future research could address this issue by collecting responses from individuals with roles and responsibilities that directly relate to strategic planning, digital transformation, and AI implementation.

Another limitation concerns the measurement of the study constructs. Some measures were newly developed or adapted from existing scales, and further validation is required to confirm their applicability across different contexts. Moreover, the use of the same sample for both exploratory factor analysis (EFA) and confirmatory factor analysis (CFA) may have affected the robustness of the measurement model. Future research should validate the measurement instruments using independent samples to provide stronger evidence of construct validity and reliability.

Finally, this study did not examine the direct relationship between strategic foresight and students’ perceptions of institutional agility or the direct influence of students’ perceptions of institutional agility on AI readiness. Although the findings indicate that students’ perceptions of institutional agility plays an important mediating role, further investigation of these direct relationships would provide a deeper understanding of how organizations transform future-oriented thinking into AI preparedness. Future studies should therefore explore these relationships using more rigorous research designs, including longitudinal, multi-source, and multi-institutional approaches, to generate stronger evidence and enable more meaningful institution-level conclusions.

## Data Availability

The raw data supporting the conclusions of this article will be made available by the authors, without undue reservation.
